# Supplementary material for: Common helical V1V2 conformations of HIV-1 Envelope expose the α4β7 binding site on intact virions
Source: Nat Commun. 2018 Oct 26;9:4489. doi: 10.1038/s41467-018-06794-x (PMC6203816; doi:10.1038/s41467-018-06794-x)
Supplement: Supplementary file 2 — Supplementary Information [file 41467_2018_6794_MOESM2_ESM.pdf]

<u>Reviewers' comments:</u>

Reviewer #1 (Remarks to the Author):

This is an excellent paper describing two new human monoclonal antibodies (mAbs) specific for a region in the C strand of the V2 region of the HIV-1 gp120 envelope glycoprotein that can assume two different conformations: that of a beta-strand and an alpha-helix. The two new mAbs are the first ones to be isolated from an HIV-infected individual that recognize the helical conformation. This work confirms and extends data that suggested these alternative conformation, provides new and useful reagents, and provides a structural explanation for how Abs of this specificity could participate in protection from HIV infection.

Two major suggestions:
--It is important to include a paragraph in the Introduction and/or the Discussion that cites that previously published structure- and computational-based data generated the hypothesis of alternative helical and beta-strand conformations for the C-strand of V2 (Spurrier et al., J. Virol., 2014) and to also stress in this context that this model has already received strong support on the basis of structural studies (cited and shown in the manuscript) with mAbs CH58 (V2p), 830A (V2i) and PG9 (V2q).

--In the Introduction, only one short sentence at the beginning of the second paragraph refers to a single paper about the role of an adhesion molecule involved in HIV binding to cells. However, there is a rather large literature, starting in 1989 (Hildreth et al., Science) that ought to be at least briefly summarized to highlight the fact that it is well established that various adhesion molecules are known to play a role in HIV/cell interactions.

Additional suggestions that would improve this excellent paper:

Abstract
Lines 20ff: Language should be more precise:
-- “Antibodies blocking this interaction… are correlated with moderate efficacy reported from the RV144 HIV-1 vaccine trial.” This is not correct. The correlation in RV144 was with Abs that bound to V1V2-gp70 which can bind to both V2p and V2i Abs, so the correlation with Abs blocking a4b7 was not established in RV144.
--“…$\alpha 4\beta 7$-blocking antibodies recognize diverse V2 conformations”. In terms of the results reported, V2 can assume diverse conformations, but the Abs that block a4b7 recognize one (helical) conformation.

Line 29: The conclusion stated at the end of the abstract is that V2p Abs play “a functional role for non-native Envelope on virion surfaces…” It is worth adding here that it also recognizes the same structure on the surface of infected cells.

Introduction
Line 47 (and throughout): “junk” Env should perhaps be replaced by “non-functional” or “aberrant” Env

Lines 66-68: The Abs that bind to V2 and block α4β7 binding were not identified as a correlate of reduced infection risk in the RV144 HIV-1 vaccine trial. See above for correction.

Lines 71-72: In referring to the 5-stranded structure of V2, only the citation for Kwon et al (ref # 21) is listed, but the reference #26 (Pan et al) should also be cited here.

Line 75ff: The authors use the designation of V2 Ab classes V2p, V2i and V2q. This nomenclature was first described in 2014 and should be cited (Spurrier B et al., J. Virol., 2014).

Results

Lines 128 and through-out: the authors describe the V2p epitope as a "helix-loop" conformation, but then refer to an alpha-helix and extended coil. This is confusing. They seem to use the terms "loop" and "extended coil" interchangeably. The language needs to be clarified. Extended coil is more consistent with the structures shown in Fig 1E.

Line 231 and Supplementary Table I. The authors refer to this table as showing ELISA data with a V2 peptide and V1V2-1FD6 iterations, but the ELISA data themselves are only described in terms of + and – signs. However, on line 231 they refer to the relative binding of different mAbs to various constructs. The authors should add a supplementary figure showing these ELISA data with titration curves for selected pairs of mAbs and peptide/scaffolded molecules.

Lines 292-3: The data in Figure 5A and description in the text that V2q and V2i mAbs recognize different surfaces of V2 was previously described and should be cited (LM Mayr et al., PLoS ONE, 2013)

Tables and Figures

Suppl. Table 1. "V2* length": what does the asterisk denote? Also, the table should not be titled Candidate antigen screen by ELISA since the least amount of data in the table refer to ELISA results.

Fig 3: (B) The legend describes the ligand for RPMI8866 cells as "92TH023 peptides". Multiple peptides? If so, this includes what? (C) Are peptides linear or cyclic?

Fig.4 and text, line 240): Figure 4 refers to the "variable" loops 1 and 2, but in the text these are referred to as "hypervariable" loops. This terminology is confusing and imprecise. As the authors cite, V1V2 forms a conserved beta-barrel, therefore V1 and V2 has a conserved structure which, in fact, reflects relative conservation in amino acid residues at specific positions. There are, however, regions within V1 and within V2 which are hypervariable. Hypervariable regions within V1 and V2 were described in a paper cited by the authors (Pan et al., 2015) and were referred to there as "strand connecting loops/A-B" (SCLAB) and SCLBC, respectively. Use of this terminology may avoid the confusion.

Figure 6: (A) It would be useful to show binding data with mAbs known to stain infected cells to indicate the proportion of cells that are expressing the V2p epitope. While relative binding to virus particles is shown in panel B, this doesn't provide the information about proportion of infected cells binding V2p mAbs. The conformation of Env on infected cells may be different from that on virions, and the data in B were probably generated with saturating conditions and so do not provide information about relative binding of the different mAbs.

Suppl. Fig 2. (A) It would be useful to include in the text the percentage of the buried surfaces areas of the peptides with the two mAbs. (B) This figure is very confusing and hard to read. The legend needs to explain how the colors in each circle were determined and what they represent. For example, the circle for 168 with the mAb 3D/CAP45 V2 peptide is yellow and purple, but purple is a color associated with mAb 16H, not 3D.

Discussion
Lines 344-5: Again, the statement that V2p Abs were correlated with reduced risk of infection in RV144 is incorrect.

Line 410-2: "The V1V2 antigens described here were complexed to V2p antibodies prior to deglycosylation…" This is confusing in that the authors describe the V1V2-1FD6 molecules as being deglycosylated in the Methods. Please specify the figure that shows this result and/or clarify in the text.

Line 422: What is meant by "aberrant forms of gp160" that preferentially adopt an α-helical V2 conformation. Is this a reference to the "junk" Env? Why are the authors talking about gp160 and not gp120?

Methods
Are all V2 peptides used linear or cyclic? Only the sequence of 92TH023 peptide is given. The sequence of other peptides (BG505) should be shown. For ELISA, were they tagged with a linker?

Grammar: line 22ff: α4β7-blocking antibodies recognize diverse V2 conformations, and current structural data suggests their binding site is occluded in prefusion envelope trimers.

Lines 366-8: the functional significance of the β-barrel conformation has been known for some time, but an understanding of the role of helical V1V2 ___ been limited by the absence of V2p antibodies that contact appropriate immunotypes…

"Alternate forms" should be "alternative forms"

Figure 2: IGHV-51 V2p mAbs defines a binding class…

Line 705: should this read 239T cells or 293T cells?

Line 779: …anionic patch the that clasps…

- Susan Zolla-Pazner

Reviewer #2 (Remarks to the Author):

The manuscript by Wibmer et al described two new human antibodies targeting the V2p epitopes of HIV-1 gp120. The results are likely to be of interest to the broad readership of Nature Communications. Addressing the following comments should further improve an otherwise excellent manuscript.
1. "Junk" Env added a negative connotation, contradicting the manuscript's conclusion.
2. The Rwork/Rfree for CAP228-16H/CAP228 V1V2-1FD6 complex were a bit high; the authors may want make an effort to improve the refinement. The authors should make it clear if all V1V2 residues of this complex were observed in the densities, and give more details about the structure.
3. Too much discussion in the Results section.
4. The primary and secondary integrin binding sites were only suggested, not proven.
5. Line 217: The epitope of 697D is very different from that of 830A and it should not be included here.
6. The numbers in the x-axis of Figure 2B are strange (10^-0.5=?).

Wibmer et al. 2018 (A common helical V1V2 conformation of the HIV-1 Envelope exposes the α4β7 binding site on intact virions)

Response to Reviewers

REVIEWER 1

Major suggestions

It is important to include a paragraph in the Introduction and/or the Discussion that cites that previously published structure- and computational-based data generated the hypothesis of alternative helical and beta-strand conformations for the C-strand of V2 (Spurrier et al., J. Virol., 2014) and to also stress in this context that this model has already received strong support on the basis of structural studies (cited and shown in the manuscript) with mAbs CH58 (V2p), 830A (V2i) and PG9 (V2q).

We have expanded the third paragraph in the Introduction to specifically cite previous structural and computational work suggesting that the V2 region of Env might switch from a β-strand to more helical conformations when freed of oligomeric constraints (lines 101 – 104, Spurrier et al., 2014 and Aiyegbo et al., 2017). Line 443 - 446 in the Discussion now also references these previous publications.

This paragraph also cites the papers that collectively provide support for these data by determining crystal structures for V2p (Liao et al., 2013), V2i (Pan et al., 2015), and V2q (Gorman et al, 2016, Cale et al., 2017, Wang et al., 2017, McLellan et al., 2011, Lee et al., 2017, Liu et al., 2017, and Rantalainen et al., 2018) HIV-1 antibodies, as well as a recent review comparing these various structures (Moore et al., 2017).

In the Introduction, only one short sentence at the beginning of the second paragraph refers to a single paper about the role of an adhesion molecule involved in HIV binding to cells. However, there is a rather large literature, starting in 1989 (Hildreth et al., Science) that ought to be at least briefly summarized to highlight the fact that it is well established that various adhesion molecules are known to play a role in HIV/cell interactions.

We have expanded the second paragraph of the Introduction to more generally refer to non-essential HIV-1 adhesion receptors like LFA-1 (Hildreth et al., 1989, Orentas et al., 1993, Ugolini, Mondor, and Sattentau, 1999), and DC-SIGN (Geijtenbeek et al., 2000) on lines 56 – 62.

Minor suggestions for the Abstract

Lines 20: Language should be more precise: "Antibodies blocking this interaction… are correlated with moderate efficacy reported from the RV144 HIV-1 vaccine trial." This is not correct. The correlation in RV144 was with Abs that bound to V1V2-gp70 which can bind to both V2p and V2i Abs, so the correlation with Abs blocking a4b7 was not established in RV144.

We have changed this line to read "V1V2-targeted antibodies were correlated with the moderate efficacy reported from the RV144 HIV-1 vaccine trial".

Line 23: "…α4β7-blocking antibodies recognize diverse V2 conformations". In terms of the results reported, V2 can assume diverse conformations, but the Abs that block a4b7 recognize one (helical) conformation.

We agree but note that CH59 (which can also block α4β7 binding to limited Y173 containing envelopes) recognizes a slightly difference helical conformation than CH58. This sentence now reads: "Monoclonal α4β7-blocking antibodies recognize two slightly different helical V2 conformations…".

Line 29: The conclusion stated at the end of the abstract is that V2p Abs play "a functional role for non-native Envelope on virion surfaces…" It is worth adding here that it also recognizes the same structure on the surface of infected cells.

Thank you. This point has been added to the concluding sentence on line 31.

Minor suggestions for the Introduction

Line 47 (and throughout): "junk" Env should perhaps be replaced by "non-functional" or "aberrant" Env

Line 47-49 has been altered to read "… also erroneously referred to as 'junk' Env".

The term 'junk Env' has also been replaced with 'aberrant Env' throughout the rest of the document.

Lines 66-68: The Abs that bind to V2 and block α4β7 binding were not identified as a correlate of reduced infection risk in the RV144 HIV-1 vaccine trial. See above for correction.

Now line 74 – 75, this has been changed to read: "antibodies that bind to V1V2 were identified as a correlate of reduced infection risk in the RV144 vaccine trial." and "Antibodies isolated from RV144 vaccine recipients were shown to block the binding of V2 to α4β7.", to clarify that these are two separate points.

Lines 71-72: In referring to the 5-stranded structure of V2, only the citation for Kwon et al (ref # 21) is listed, but the reference #26 (Pan et al) should also be cited here.

Pan et al., 2015 has been added (now reference 25) as the first paper to define the 5-stranded β-barrel on line 79.

Line 75: The authors use the designation of V2 Ab classes V2p, V2i and V2q. This nomenclature was first described in 2014 and should be cited (Spurrier B et al., J. Virol., 2014).

We apologize for the oversight, and have now included a citation to Spurrier et al., 2014 on line 87 – 88 for defining V2p, V2i, and V2q antibody binding modalities.

Minor suggestions for the results

Lines 128 and through-out: the authors describe the V2p epitope as a "helix-loop" conformation, but then refer to an alpha-helix and extended coil. This is confusing. They seem to use the terms "loop" and "extended coil" interchangeably. The language needs to be clarified. Extended coil is more consistent with the structures shown in Fig 1E.

All references to a helix-loop conformation of V2 have been replaced with helix-coil throughout the text and Figures.

Line 231 and Supplementary Table I. The authors refer to this table as showing ELISA data with a V2 peptide and V1V2-1FD6 iterations, but the ELISA data themselves are only described in terms of + and – signs. However, on line 231 they refer to the relative binding of different mAbs to various

constructs. The authors should add a supplementary figure showing these ELISA data with titration curves for selected pairs of mAbs and peptide/scaffolded molecules.
Supplementary Table 1 has now been merged with Supplementary Figure 1A, and titration curves have been added for CAP228-16H and PG9.

Lines 292-3: The data in Figure 5A and description in the text that V2q and V2i mAbs recognize different surfaces of V2 was previously described and should be cited (LM Mayr et al., PLoS ONE, 2013)
This citation has been added (line 332).

Minor suggestions for the tables and figures

Suppl. Table 1. "V2* length": what does the asterisk denote? Also, the table should not be titled Candidate antigen screen by ELISA since the least amount of data in the table refer to ELISA results.
This table has now been incorporated into Supplementary Figure 1 and has been retitled: "Properties of candidate antigens screened by ELISA for CAP228-16H cocrystal studies. Asterisks have been used to further define the regions included in V1 (positions 132-156) and the hypervariable region of V2 (positions 182-190).

Fig 3: (B) The legend describes the ligand for RPMI8866 cells as "92TH023 peptides". Multiple peptides? If so, this includes what? (C) Are peptides linear or cyclic?
Only one cyclic peptide was used, and this has been corrected in the Figure legend (line 779).

Fig.4 and text, line 240): Figure 4 refers to the "variable" loops 1 and 2, but in the text these are referred to as "hypervariable" loops. This terminology is confusing and imprecise. As the authors cite, V1V2 forms a conserved beta-barrel, therefore V1 and V2 has a conserved structure which, in fact, reflects relative conservation in amino acid residues at specific positions. There are, however, regions within V1 and within V2 which are hypervariable. Hypervariable regions within V1 and V2 were described in a paper cited by the authors (Pan et al., 2015) and were referred to there as "strand connecting loops/A-B" (SCLAB) and SCLBC, respectively. Use of this terminology may avoid the confusion.
The terms "strand connecting loop AB or C'D" are unfortunately no longer relevant for the helical structures presented in this manuscript, since the hypervariable loop regions no longer connect β-strands. We agree that the hypervariable and conserved segments of 'Variable region 2 (or V2)' could be better defined. On lines 81 – 87 of the Introduction, we have included a description of the V1V2 domain referring specifically to the hypervariable loop component of V2, defined as including residues 182-189. Throughout the manuscript and Figures we have referred to this loop as the "hypervariable loop region of V2". V1 is for the most part always hypervariable and is referred to as just V1.

Figure 6: (A) It would be useful to show binding data with mAbs known to stain infected cells to indicate the proportion of cells that are expressing the V2p epitope. While relative binding to virus particles is shown in panel B, this doesn't provide the information about proportion of infected cells binding V2p mAbs. The conformation of Env on infected cells may be different from that on virions, and the data in B were probably generated with saturating conditions and so do not provide information about relative binding of the different mAbs.
It is difficult to calculate a consistent number for the proportions of Env bound by a particular class of antibody such as V2p-only. This is because the expression level and extent of glycosylation vary between experiments, but also because simple staining experiments do not give a clear indication as to whether

cells/virions are expressing one/both types of Env on their surfaces. In the case of V2p antibodies this might be further complicated by the possibility that V1V2 might be able to transition between these two alternative conformations. Because of these additional factors we do not feel comfortable reporting relative proportions, but rather chose to simply report that V2p epitopes are detectable on cell/virion surfaces, and therefore on physiologically relevant membrane associated gp160 proteins.

Suppl. Fig 2. (A) It would be useful to include in the text the percentage of the buried surfaces areas of the peptides with the two mAbs. (B) This figure is very confusing and hard to read. The legend needs to explain how the colors in each circle were determined and what they represent. For example, the circle for 168 with the mAb 3D/CAP45 V2 peptide is yellow and purple, but purple is a color associated with mAb 16H, not 3D.

The contact surfaces area between V2 and the V2p mAb heavy chains has been added on lines 167-170 and interacting light chains surfaces are compared between lines 203-206. The erroneous purple for position 168 has been corrected, and a more detailed figure legend has been provided.

<u>Minor suggestions for the Discussion</u>

Lines 344-5: Again, the statement that V2p Abs were correlated with reduced risk of infection in RV144 is incorrect.

Now line 381 – 382, as above this has been corrected to V1V2-targeted antibodies.

Line 410-2: "The V1V2 antigens described here were complexed to V2p antibodies prior to deglycosylation…" This is confusing in that the authors describe the V1V2-1FD6 molecules as being deglycosylated in the Methods. Please specify the figure that shows this result and/or clarify in the text.

Scaffolds were deglycosylated with EndoH after they had been captured by and thus complexed to antibody prebound to protein A beads. This has been clarified in the Methods section (lines 496-500) and removed from the Results to avoid confusion.

Line 422: What is meant by "aberrant forms of gp160" that preferentially adopt an α-helical V2 conformation. Is this a reference to the "junk" Env? Why are the authors talking about gp160 and not gp120?

As per the above correction, all references to 'junk' Env have now been replaced with <u>aberrant Env</u>. On lines 471-472 we have clarified that aberrant Env includes "gp160 protomers still anchored on the surfaces of HIV-1 virions or infected cells…" as suggested by our infected cell FACS staining and pseudovirus ELISA binding assays.

<u>Minor suggestions for the Methods</u>

Are all V2 peptides used linear or cyclic? Only the sequence of 92TH023 peptide is given. The sequence of other peptides (BG505) should be shown. For ELISA, were they tagged with a linker?

A single peptide sequence was used for each wild-type strain. For x-ray crystallography these were short linear peptides, while binding / competition assays were performed with slightly longer, biotinylated, cyclic V2 peptides. This has been clarified on lines 504 – 505, 557, and 779. The BG505 V1V2 sequence has been added to Figure 1D.

<u>Minor suggestions for the Grammar</u>

Line 22: α4β7-blocking antibodies recognize diverse V2 conformations, and current structural data suggests their binding site is occluded in prefusion envelope trimers.
This sentence has been modified.

Lines 366-8: the functional significance of the β-barrel conformation has been known for some time, but an understanding of the role of helical V1V2 ___ been limited by the absence of V2p antibodies that contact appropriate immunotypes…
This has been corrected on line 404.

"Alternate forms" should be "alternative forms"
This has been corrected throughout the document.

Figure 2: IGHV-51 V2p mAbs defines a binding class…
This has been corrected to IGHV5-51.

Line 705: should this read 239T cells or 293T cells?
Corrected to 293T cells on line 772.

Line 779: …anionic patch the that clasps…
This sentence has been corrected by removing the word "the", line 866.

REVIEWER 2

1. **"Junk" Env added a negative connotation, contradicting the manuscript's conclusion.**
We agree, and this has been changed to aberrant Env throughout the document.

2. **The Rwork/Rfree for CAP228-16H/CAP228 V1V2-1FD6 complex were a bit high; the authors may want make an effort to improve the refinement. The authors should make it clear if all V1V2 residues of this complex were observed in the densities, and give more details about the structure.**
The difference between Rwork/Rfree was improved to 0.24/0.28 (shown in Table 1), but overall the **[Editorial note: the authors report that this was subsequently improved to 0.22/0.27.]** model does suffer from the inherent plasticity of V1V2 when scaffolded onto the 1FD6 protein. To further illustrate this we have added additional data to Supplementary Figure 3 showing the 2Fo-Fc density maps for the ordered V2p epitope, as well as the more disordered V1 and V2 hypervariable loop regions, and described in the text those amino acids at the apex of the V2 hypervariable loop that did not have substantial observed electron density due to loop flexibility on lines 275. We have also included more details about this structure on lines 184 – 188, 261 – 305, and reorganized the paragraph accordingly.

3. **Too much discussion in the Results section.**
We have moved discussion points on the mutability of position 28 in the IGVH5-51 gene (line 180 – 182), the inability of β-strand recognizing antibodies to block α4β7 binding (lines 237 – 240), and the potential role for the CDR-H3 lengths of IGHV5-51 V2p antibodies (lines 298 – 305) to the Discussion lines 413 – 417, 435 – 442, and 450 – 456 respectively.

4. **The primary and secondary integrin binding sites were only suggested, not proven.**
This has been changed throughout the document and figures to refer to the potential secondary binding determinant.

5. Line 217: The epitope of 697D is very different from that of 830A and it should not be included here.

We have removed any reference to antibody 697D.

6. The numbers in the x-axis of Figure 2B are strange (10^-0.5=?).

The x-axis labels for Figure 3B have been corrected.
